# Techno-Economic Comparison Based on Experimental Setup of Spherical and Flat Photovoltaics with IoT Monitoring System

**DOI:** 10.3390/s26113499

**Published:** 2026-06-02

**Authors:** Ahmed Badawi, Claude Ziad El-Bayeh, I. M. Elzein, Walid Alqaisi, Azad Ashraf, Vesna Palikuca, Mazhar Hasan-Zia

**Affiliations:** Department of Electrical Engineering, University of Doha for Science and Technology, Doha P.O. Box 24449, Qatar; claude.elbayeh@udst.edu.qa (C.Z.E.-B.); azad.ashraf@udst.edu.qa (A.A.);

**Keywords:** spherical photovoltaic, spherical solar, Internet of Things (IoT), solar energy, integrated reflector, experimental setup

## Abstract

**Highlights:**

**What are the main findings?**

- The spherical photovoltaic (SPV) system with an integrated reflector achieved 32.2% higher energy output, operated at 8–12 °C cooler, and maintained a more stable voltage than a conventional flat PV panel.
- The SPV design reduced dust accumulation by 27%, required ~35% less installation area per watt, and benefited from reflector-assisted power gains of 14.8–39.7%.

**What are the implication of the main finding?**

- The SPV system provides a compact, efficient, and omnidirectional solution suitable for urban and extreme-environment solar energy harvesting.
- IoT-based monitoring enables real-time performance tracking and automated alerts, supporting intelligent energy management and preventive maintenance.

**Abstract:**

This paper presents an experimental investigation of a spherical photovoltaic (SPV) system enhanced with an integrated paraboloid reflector and monitored via an Internet of Things (IoT) platform. The SPV’s omnidirectional geometry enables improved light absorption from multiple angles, maximizing energy capture throughout the day and under diverse weather conditions, particularly in extreme climates such as in Qatar. A prototype was developed using photovoltaic cells mounted on a 30 cm diameter spherical frame, paired with a reflector constructed from Styrofoam covered with mini glass mirrors. Performance was benchmarked against a conventional flat photovoltaic (FPV) panel with an equal number of cells. Real-time IoT monitoring captured voltage, temperature, and irradiance data, enabling precise performance evaluation. Results demonstrate that the SPV system achieved a 32.2% higher weekly energy output than the FPV panel, with reflector-assisted gains ranging from 14.8% to 39.7%. The SPV operated at 8–12 °C cooler, producing more stable voltage outputs (24–28 V vs. 17–25 V). Additionally, the design reduced dust accumulation by 27% and required ~35% less installation area per watt. IoT integration facilitated automated monitoring and alerts for critical conditions such as overheating (>50 °C) or voltage drops (<12 V). These findings highlight the SPV system as a compact, efficient, and intelligent solution for next-generation solar energy harvesting in urban and extreme-environment applications.

## 1. Introduction

### 1.1. Background and Motivation

Globally, researchers are actively seeking sustainable and clean energy solutions that minimize environmental impact and mitigate climate change. Among these, solar energy stands out as a promising, renewable source that meets these critical criteria [1,2,3,4]. Solar energy is the most abundant and widely accessible energy source on Earth. Among renewable energy technologies, photovoltaic (PV) power generation has attracted significant global attention and has achieved the fastest adoption, owing to its technological maturity, scalability, and versatility [5,6,7,8,9,10]. Due to the technology’s low cost, it is attracting investment in power production technologies [11,12]. The globally installed PV capacity is expected to surpass that of nuclear, wind, and gas power [13,14]. Solar energy is widely available almost everywhere. However, the efficiency of using solar radiation to generate electricity is currently low because the direction of the sun’s beam varies depending on the time of day and season [15,16,17]. Therefore, improving the efficiency of using solar radiation to generate electricity is crucial [18]. Most commercially available solar panels are made from silicon, which has a maximum efficiency limit of 33% [19,20]. Typically, photovoltaic panels are installed facing the sun on the roof of a house or in a nearby open space [21,22,23]. To generate the maximum energy possible, a solar panel needs to be oriented perpendicular to the light source. Since the sun moves throughout the day and year, a solar panel needs to track the sun’s movement to generate the most power possible. Using a tracking mechanism to keep the panel aligned orthogonally with the light source is one of the best available solutions [24,25,26]. One of the most effective ways to increase solar panel efficiency is to use a solar tracking system, which adjusts the azimuth and zenith of the panels’ orientation [12,27]. The panels continuously face the sun due to the tracker, resulting in a larger increase in electricity production because the electrical energy produced by a PV system is directly proportional to the solar irradiation received in the generator panel and the installed nominal power [28,29]. However, solar trackers have their limitations, such as the higher initial installation cost compared to traditional fixed-tilt systems and higher maintenance costs [30,31]. Additionally, solar tracker systems cannot be implemented in locations with high winds or inclement weather, which can affect the movement and durability of the gears and motors in the system [32,33]. To address these concerns, spherical photovoltaic (SPV) panels could be an alternative to the traditional flat photovoltaic (FPV) panels, which will be the subject of investigation in this paper.

Particularly in Qatar, solar energy has strong advantages due to the country’s very high solar irradiance, long sunshine duration, and clear skies, which make it highly suitable for efficient photovoltaic power generation and large-scale deployment [34]. Solar PV aligns well with Qatar’s daytime peak electricity demand, supports energy diversification, and helps reduce natural gas consumption and carbon emissions [35]. However, its performance is limited by harsh environmental conditions, particularly high ambient temperatures that reduce PV efficiency [36,37,38], frequent dust and sand accumulation that cause significant soiling losses, and water scarcity that complicates module cleaning and maintenance. These challenges make Qatar a demanding but valuable environment for developing and validating advanced and resilient photovoltaic system designs.

### 1.2. Brief Literature Review on Spherical Solar Panels

In recent years, researchers have focused on enhancing photovoltaic (PV) system performance and efficiency by modifying structural designs and surface characteristics of solar panels. Researchers in reference [39] systematically evaluated curved PV configurations under varying climatic conditions in Palembang, Indonesia, comparing data from the dry season and rainy season. The concave design demonstrated optimal results during dry periods, achieving a peak current of 20.27 W and 13.14% efficiency, outperforming flat and convex layouts. Notably, convex configurations still surpassed traditional flat panels in power output and efficiency, highlighting the potential of curved geometries to boost solar energy capture. Further innovation emerged in [40]: a flexible circular solar cell using monocrystalline silicon achieved 19% efficiency without requiring sun-tracking mechanisms. Incorporating a waste-reduction approach, this design outperformed standard 4 cm flat cells by 14.8% in immediate power generation, with gains soaring to 39.7% when paired with reflective surfaces like sand or white paper. Its angled orientation also minimized dust buildup, offering practical benefits for long-term solar applications. While studies have investigated reflective surfaces in tandem with spherical or curved PV arrays to amplify energy yields, the integration of IoT technologies for real-time performance optimization remains underexplored, presenting a critical area for future research. Recent research into curved photovoltaic (PV) designs, such as the hemispherical module analyzed in [41], has revealed challenges in scalability and a gap in real-time performance monitoring. The study in [41] conducted a systematic comparison of flexible curved solar modules across flat, cylindrical, and hemispherical surfaces. Results indicated that flat surfaces exhibited the lowest fill factor (0.84), while cylindrical and hemispherical configurations showed marginally higher values of 0.88 and 0.84, respectively. Power output peaked at 61.14 W for hemispherical surfaces, surpassing cylindrical (59.8 W) and flat (57.8 W) designs. Authors in [42] conducted an experimental study on semi-flexible monocrystalline solar panels configured in flat, convex, and concave arrangements to assess their power output and efficiency under tropical conditions. The results showed that the concave configuration consistently outperformed the others, achieving a maximum power output of 20.27 W and an efficiency of 13.14% during the dry season. The study highlighted the potential of arched panel settings, particularly concave arrangements, for dynamic applications such as agricultural robots. However, limitations included the short duration of testing, single-location data, and the absence of cost and mechanical stability considerations, which may affect broader applicability. Authors in [43] proposed a novel nature-inspired spherical silicon solar cell capable of three-dimensional light harvesting by capturing direct, diffuse, and background-reflected sunlight. Fabricated using a corrugation technique that preserves the electrical performance of monocrystalline silicon, the spherical design significantly enhanced power output—up to 101% more than flat cells of the same ground area under optimal reflective conditions. Additionally, the spherical cell demonstrated superior thermal management, with up to 31.6% lower average operating temperatures, and reduced dust accumulation, due to its geometry. However, limitations of the study include its reliance on controlled laboratory conditions using solar simulators and limited environmental variability, which may not fully represent real-world performance across diverse climates and deployment scenarios. To the best of our knowledge, spherical solar panels are not integrated with parabolic reflectors and are not supervised with IoT-based monitoring system for performance optimization, which will be the subject of this paper.

### 1.3. Contributions

In this paper, authors conducted an experimental setup of our proposed spherical photovoltaic panel prototype with a parabolic reflector in order to increase energy yield and reduce the land footprint. The SPV system is integrated with IoT-enabled real-time monitoring system to track weather conditions such as temperature and solar radiation, and measure the output voltage, current and power from the SPV system. The SPV system is compared to a flat PV system which has the same number of solar cells. This integration, thus, mitigates the performance challenges associated with static spherical designs and offers a progressive approach for large-scale deployment.

### 1.4. Paper Organization

The paper is organized as follows: In Section 2, authors present the mathematical modeling of the spherical photovoltaic system with a paraboloid reflector. Section 2 also proposed the maximum theoretical efficiency threshold of the SPV. In Section 3, the designed and implemented SPV system with an integrated reflector is presented. Results and discussions are presented in Section 4, in which authors compared both systems under real weather conditions in Doha, Qatar. Finally, the conclusion and future work are presented in Section 5.

## 2. Mathematical Modeling of an SPV Panel with a Paraboloid Reflector

### 2.1. Mathematical Modeling of a Spherical Photovoltaic (SPV) Panel

In this section, authors present the mathematical modeling of the spherical photovoltaic panel that will be used to determine the incident power on the SPV and the output power. The mathematical equation of a sphere in a 3D space is presented in Equation (1), where $x_{1}$, $y_{1}$, and $z_{1}$ are the center of the sphere on the corresponding axis x, y and z.

(1) $${\left(x-x_{1}\right)}^{2}+{\left(y-y_{1}\right)}^{2}+{\left(z-z_{1}\right)}^{2}=R^{2}$$

Since the SPV is not a perfect sphere, its shape can be categorized as a Platonic solid in which its volume is expressed as in Equation (2), where a is the edge length of a face, p is the number of edges of each face (e.g., four for the case of a square), F is the number of faces (e.g., the number of solar cells on the sphere), and θ is the dihedral angle between two intersecting faces. For approximation, Equation (3) is used to determine the volume of the SPV panel, in which R is the radius of the sphere, and α is the approximation factor ($\alpha_{V}<1$) between the theoretical volume expressed in Equation (2) and our prototype.

(2) $$V_{Platonic}^{SPV}=\frac{a^{3}}{24}\cdot \cot^{2}\left(\frac{\pi }{p}\right)\cdot F\cdot p\cdot \tan\left(\frac{\theta }{2}\right)$$

(3) $$V_{S}^{SPV}=\frac{4}{3}\cdot \pi \cdot \alpha_{V}\cdot R^{3}$$

The area of the Platonic solid such as SPV panel is expressed by Equation (4). However, for approximation, Equation (5) is used, where $\alpha_{A}$ is the approximation factor ($\alpha_{A}<1$) between the theoretical area expressed in Equation (4) and our prototype.

(4) $$A_{Platonic}^{SPV}=F\cdot p\cdot \cot\left(\frac{\pi }{p}\right)\cdot \frac{a^{2}}{4}$$

(5) $$A_{S}^{SPV}=4\cdot \pi \cdot \alpha_{A}\cdot R^{2}$$

Since the solar cells do not fit the sphere completely, there will be unused areas on the surface $A_{unused}^{SPV}$ which cannot generate electricity. Therefore, the used area of the SPV panel is expressed by Equation (6).

(6) $$A_{S,used}^{SPV}=A_{S}^{SPV}-A_{unused}^{SPV}$$

**Figure 1:**
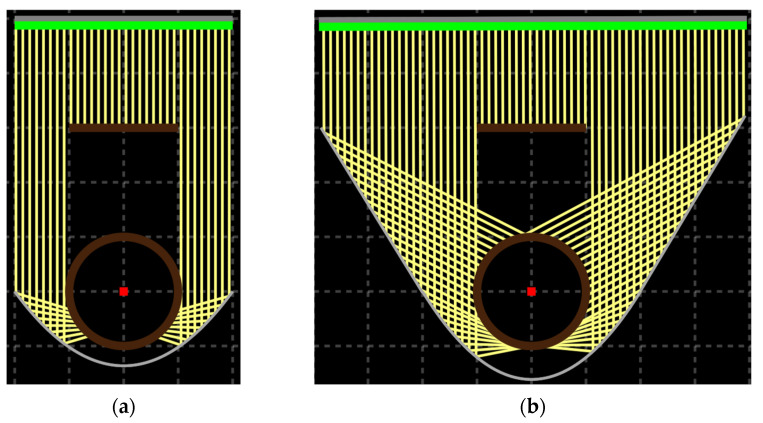
Indirect solar radiation that covers different areas on the SPV: (**a**) small area of the lower hemisphere, and (**b**) all the sphere’s surface including the upper and lower hemispheres.

Practically, the SPV receives two types of solar radiation. The first type is direct solar radiation (DSR) that hits the first hemisphere (e.g., north), while the second is indirect solar radiation (ISR) which is reflected by a reflector and received by the second hemisphere (e.g., south). Hence, there are two areas to be determined based on the direct and indirect solar radiation received by the SPV panel. The area of the SPV where the direct solar radiation hits the surface ($A_{S,SDR}^{SPV}$) is expressed by Equation (7), where $\alpha_{S,SDR}^{SPV}$ is the filling factor, between 0 and 1; a value equal to 1 means that the SPV receives radiation on its half hemisphere. On the other hand, the area of the SPV where the indirect solar radiation hits the surface ($A_{S,IDR}^{SPV}$) is expressed by Equation (8), where $\alpha_{S,IDR}^{SPV}$ is the filling factor, between 0 and 1, where a value equal to 1 means that the SPV receives indirect solar radiation on all its surface, while a value equal to 0 means that there is no indirect solar radiation received by the SPV. The main reason for $\alpha_{S,IDR}^{SPV}\in [0,1]$ is that the SPV can receive indirect solar radiation from the reflector on a part or all its surface as presented in Figure 1.

(7) $$A_{S,SDR}^{SPV}=\frac{\alpha_{S,SDR}^{SPV}}{2}\cdot A_{S,used}^{SPV}$$

(8) $$A_{S,IDR}^{SPV}=\alpha_{S,IDR}^{SPV}\cdot A_{S,used}^{SPV}$$

### 2.2. Mathematical Modeling of the Paraboloid Reflector

In this section, authors present the mathematical modeling of the paraboloid reflector that will be used to reflect the solar radiation to the bottom hemisphere of the SPV. The mathematical equation of a paraboloid in a 3D space is presented by Equation (9), where $X^{T}$ is the transpose of matrix X in Equation (10), which represents the coordinate of measure points in 3D space (x, y, z), and C is the coefficient matrix as described in Equation (11). Since the reflector is limited in dimension, it is necessary to limit the coefficients in the matrix C as presented in Equation (12). In general, the paraboloid reflector is used to concentrate the solar radiation to a focal point; in the case of this paper, it will be the center of the sphere. However, since the solar cells are located on the surface of the sphere, it means that the solar radiation will spread over the solar cells with less concentration than at the focal point in order to limit the rise in temperature on these cells.

(9) $$X^{T}CX=0$$

(10) $$X=\left[\begin{array}{c} 1\\ x-x_{0}\\ y-y_{0}\\ z-z_{0} \end{array}\right]$$

(11) $$C=\left[\begin{array}{cccc} c_{11} & 0 & 0 & 0\\ c_{21} & c_{22} & 0 & 0\\ c_{31} & c_{32} & c_{33} & 0\\ c_{41} & c_{42} & c_{43} & c_{44} \end{array}\right]$$

(12) $$c_{ij}^{Min}\leq c_{ij}\leq c_{ij}^{Max}$$

**Figure 2:**
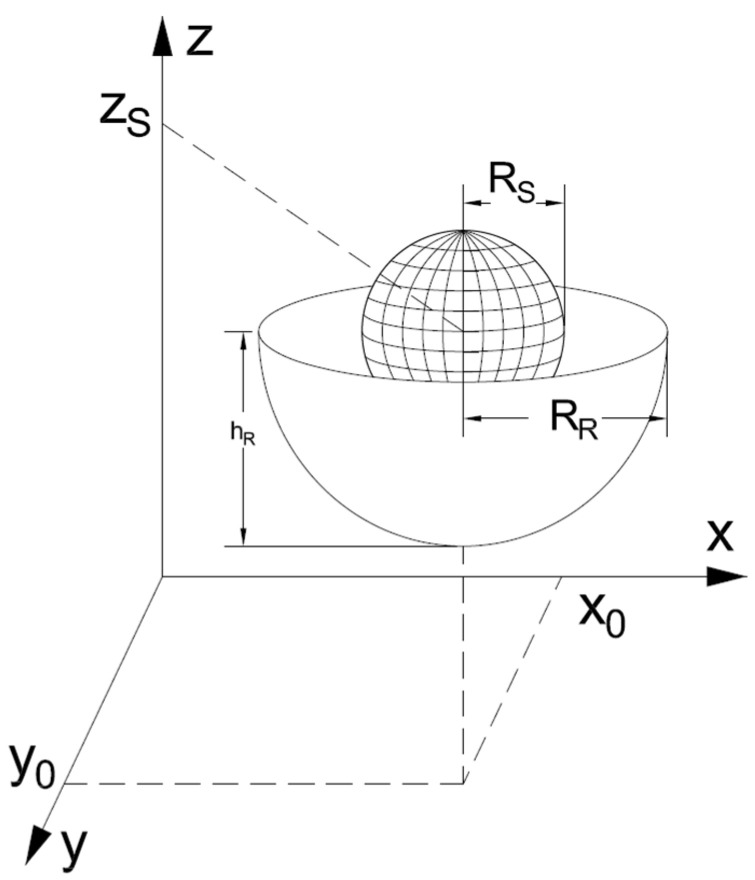
Vertical positioning of the SPV with the paraboloid reflector in 3D plane.

For a particular case, and for a vertical paraboloid that points toward the SPV on a vertical axis as presented in Figure 2, the coefficient matrix is as shown in Equation (13).

(13) $$D=\left[\begin{array}{cccc} 0 & 0 & 0 & 0\\ 0 & c_{22}=\frac{1}{a} & 0 & 0\\ 0 & 0 & c_{33}=\frac{1}{b} & 0\\ c_{41}=-\frac{1}{c} & 0 & 0 & 0 \end{array}\right]$$

### 2.3. Mathematical Modeling of the Input and Output Power and Energy

The total input power of the SPV ($P_{S,t}^{In}$) received through direct and indirect solar radiations is expressed in Equation (14), where $I_{t}^{SDR}$ is the direct solar radiation ($W/m^{2}$); $A_{S,SDR,t}^{SPV}$ is the area of the SPV which receives the direct solar radiation; $\eta_{R}$ is the efficiency of the reflector; $A_{S,ISR,t}^{SPV}$ is the area of the SPV which receives indirect solar radiation reflected by the paraboloid reflector; and $\eta_{S}^{SPV}$ is the efficiency of the received radiation on the SPV. Since the power is conserved, $I_{in}A_{In}=I_{out}A_{out}$, the received indirect solar radiation by the SPV can be expressed by Equation (15), where $R_{R}$ and $R_{S}$ are the radii of the reflector and sphere, respectively, and f is the ratio factor between the radius of the reflector and the sphere. All the parameters are functions of time since they are highly dependent on weather conditions and the solar incident angle on the SPV.

(14) $$P_{S,t}^{In}=I_{t}^{DSR}\cdot A_{S,DSR,t}^{SPV}+\left(\eta_{R}\cdot I_{t}^{ISR}\right)\cdot A_{S,ISR,t}^{SPV}\cdot \eta_{S}^{SPV}$$

(15) $$I_{t}^{ISR}=\frac{I_{t}^{SDR}\cdot \left(\pi R_{S}^{2}\left(f^{2}-1\right)\right)}{\eta_{R}\cdot A_{S,ISR,t}^{SPV}\cdot \eta_{S}^{SPV}}$$

where $R_{R}=fR_{s}$, and f>1

The output power of the SPV ($P_{S,t}^{Out}$) is the average output power for all solar cells. The efficiency ($\eta_{S}^{out}$) is highly dependent on the topology configuration of the solar cells, which is expressed by Equation (16). The input ($E_{S}^{In}$) and output ($E_{S}^{Out}$) energy of the SPV are expressed by Equations (17) and (18).

(16) $$P_{S,t}^{Out}=\eta_{S}^{out}P_{S,t}^{In}$$

(17) $$E_{S}^{In}=\sum_{t\in T}P_{S,t}^{In}\cdot \Delta t$$

(18) $$E_{S}^{Out}=\sum_{t\in T}P_{S,t}^{Out}\cdot \Delta t$$

### 2.4. Comparison Between the Footprint Area of the SPV and FPV

#### 2.4.1. Footprint Area of a Photovoltaic Module

**Figure 3:**
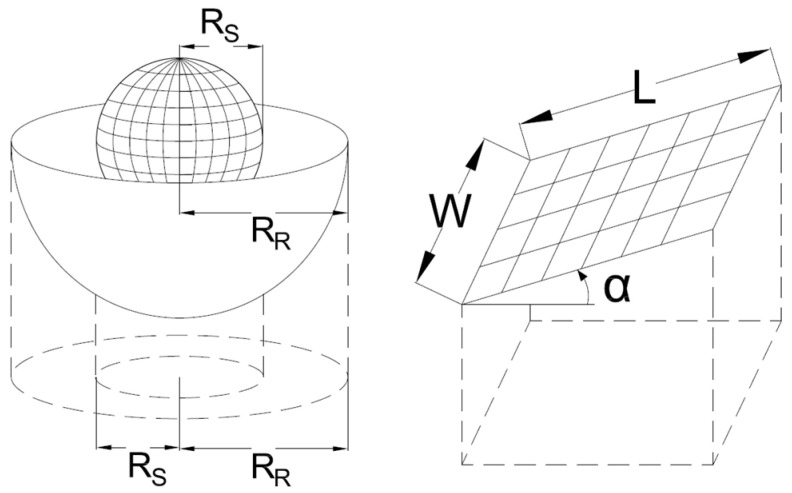
Footprint area of SPV and FPV.

The footprint area of a PV module is defined as the total physical space that this module occupies on a given surface (like a roof or the ground). For the flat photovoltaic module (FPV), the footprint area is defined by Equation (19), where w and l are the width and length of the solar panel, respectively, as in Figure 3 right side; α is the tilt angle on a flat surface; β is the ratio between the length and the width of the panel. On the other hand, the footprint area of an SPV with and without a reflector is determined by Equations (21) and (22), respectively, Figure 3 left side, where $R_{S}$ and $R_{R}$ are the radius of the footprint circle of the sphere and reflector, respectively, and $\beta_{R}$ is the ratio between the radius of the reflector and the sphere.

(19) $$A_{F}^{FPV}=\beta \cdot w^{2}\cdot cos(\alpha )$$

where

(20) l=β·w

(21) $$A_{F}^{SPV}=\pi \cdot R_{S}^{2}$$

(22) $$A_{F}^{SPV,R}=\pi \cdot {\left(\beta_{R}\cdot R_{S}\right)}^{2}$$

where

(23) $$R_{R}=\beta_{R}\cdot R_{S}\;\mathrm{For}\;\beta_{R}\geq 1$$

#### 2.4.2. Footprint Area Ratio

The footprint area ratio (FAR) is a comparative metric used to evaluate the physical space ratio between the FPV and SPV that occupies a given surface. Since FPV and SPV are being compared, both systems should have the same surface of photovoltaic cells despite the given shape. In this case, Equation (24) relates the width of the FPV to the radius of the SPV ($4\pi R_{S}^{2}=\beta w^{2}$). Thus, by substituting Equation (24) into Equation (19), the footprint area of the FPV becomes a function of the radius of the sphere and the tilt angle as shown in Equation (25).

(24) $$w=2R_{S}\sqrt{\frac{\pi }{\beta }}$$

(25) $$A_{F}^{FPV}=4\pi \cdot R_{S}^{2}\cdot cos(\alpha )$$

Therefore, the footprint area ratio is calculated according to Equation (26). In other words, if the tilt angle of the FPV is zero, and the radius of the reflector is the same as the sphere, the ratio will be equal to 4, which means that four SPVs can fit the same surface of one FPV. Therefore, energy production is theoretically quadrupled. However, in practice, the tilt angle is around 30°, and the reflector has a radius of around $\sqrt{2}R_{S}$, which results in an FAR = $\sqrt{3}$.

(26) $$FAR=\frac{A_{F}^{FPV}}{A_{F}^{SPV}}\;FAR=\frac{4\pi \cdot R_{S}^{2}\cdot \cos\left(\alpha \right)}{\pi R_{R}^{2}}\;FAR=\frac{4\cdot cos(\alpha )}{\beta_{R}^{2}}$$

#### 2.4.3. Maximum Theoretical Efficiency of the SPV

In this section, authors suppose that the same area of solar cells is used for both FPV and SPV. The solar radiation is considered to hit each solar cell perpendicularly for the FPV and the SPV in a condition where the reflector radius is $R_{R}=\sqrt{2}R_{S}$. In other words, the SPV receives the same solar radiation on all its cells. Therefore, the maximum theoretical efficiency of the SPV ($\eta_{Max}^{SPV}$) is determined by Equation (27), where $A^{FPV}$ and $A^{SPV}$ are the surface of the FPV ($\beta \cdot w^{2}$) and SPV ($4\pi R_{S}^{2}$), respectively; $\eta^{FPV}$ is the efficiency of the FPV. Hence, SPV has doubled the efficiency of the FPV. Thus, if a PV panel has an efficiency of 23%, it means that for the same surface area using a spherical PV, the efficiency is doubled to 46%. However, this theoretical efficiency cannot be reached due to many factors such as the reflection losses of the reflector, shadows of the sphere on the reflector, in cases where there is no tracking system, etc. That being said, an improvement in efficiency of between 1.2$\eta^{FPV}$ and 1.8$\eta^{FPV}$ is expected in practice.

(27) $$\eta_{Max}^{SPV}=\frac{A^{FPV}\eta^{FPV}}{A^{SPV}}=2\eta^{FPV}$$

## 3. Design and Implementation of the SPV with Integrated Reflector

The innovative spherical photovoltaic (SPV) system with integrated reflectors was designed to address the limitations of traditional flat-panel solar technologies, particularly under conditions of varying solar incidence angles. The design leverages the geometry of a sphere to maximize solar irradiance capture throughout the day and across seasons without the need for active sun-tracking systems, thereby increasing the likelihood of energy conversion regardless of the sun’s position.

### 3.1. SPV Design and Construction

**Figure 4:**
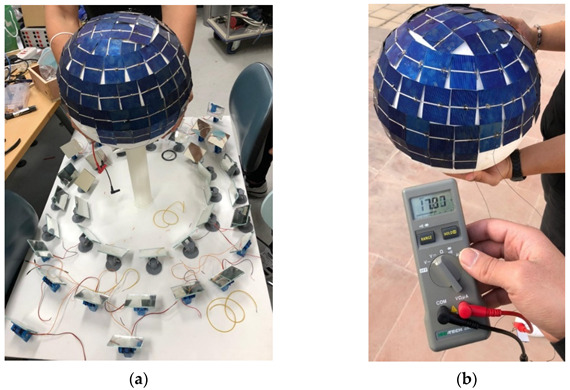
Construction of the SPV with reflectors: (**a**) prototype phase, (**b**) testing phase.

The SPV module is constructed from multiple small photovoltaic cells precisely mounted on a Styrofoam ball (30 cm of diameter), photovoltaic cells (52 mm × 78 mm, Polycrystalline silicon, 0.7 W peak), wooden shaft (30 cm), foam board, irradiance sensor, voltage sensor, temperature sensor, and a wireless transceiver (X Bee/Wifi) as presented in Figure 4. The X Bee ensures reliable system performance with higher effectiveness [44]. Photovoltaic cells are electrically interconnected in a series–parallel configuration to balance voltage and current requirements. The modularity of the cell arrangement also supports fault tolerance, allowing the system to remain partially functional even if individual cells fail.

The SPV prototype was fabricated and tested under both controlled indoor and outdoor environments. Figure 4a shows the indoor testing. The testing involves a controlled light environment using the reflector array. The alignment of the mirrors was adjusted to ensure concentrated light onto the SPV surface, simulating a scenario of enhanced irradiance. Measurements confirmed increased power output when the reflector system was active, underscoring the advantage of the integrated approach. Figure 4b shows the outdoor testing, which was conducted to evaluate the electrical performance of the SPV under real solar conditions. The system achieved an open-circuit voltage of approximately 17.8 V under direct sunlight, demonstrating the feasibility of the spherical configuration for energy harvesting. The list of components used for the installed system is described in Table 1.

### 3.2. Reflector Design and Construction

**Figure 5:**
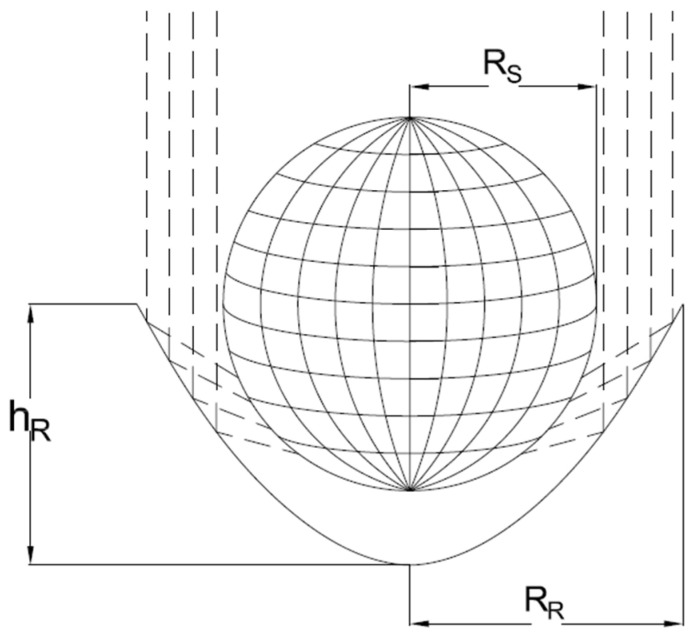
Reflector dimensions (dotted lines represent the direct and reflected solar radiation).

A critical component of the SPV system is the integration of a reflector designed to enhance light concentration on the lower part of the hemisphere of the SPV. The reflector consists of a paraboloid shape made of Styrofoam and covered with an array of mini square glass mirrors. This enhances the effective irradiance on less-exposed regions of the sphere, particularly during low-incidence periods such as early morning and late afternoon. The radius of the reflector is around 21.21 cm ($\sqrt{2}R_{S}$), and its height is around 21.21 cm from the center of the sphere as shown in Figure 5. In addition, the reflector has a hole at its bottom part, in which it helps in releasing accumulated dust to avoid loss of reflective efficiency and durability.

### 3.3. Comparison Procedure Between SPV and FPV

The same number of photovoltaic cells on flat and spherical panels are used for comparison purposes. The main reason is to compare the efficiency of both SPV and FPV based on the reading of voltage, temperature, and irradiance by sensors. The FPV is positioned, and its tilt angle is determined through experimentation, by which the maximum efficiency can be obtained.

### 3.4. Data Acquisition and Monitoring Using IoT Technology

**Figure 6:**
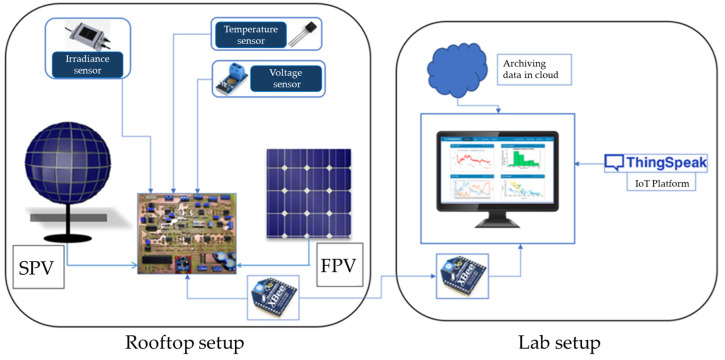
Integration of IoT technology with SPV and FPV for monitoring and data collection.

The SPV and FPV are integrated with Internet of Things (IoT) technology for comparison purposes, and to facilitate immediate monitoring of essential performance metrics, including temperature and voltage as shown in Figure 6. Internal sensors are hooked up inside the solar panels to accumulate information related to the temperature of the cells and the output voltage output. A microprocessor is used to collect data and forward it to at least one IoT imperative and to allow remote monitoring and evaluation. Such parameters, while being tracked, create a higher room for optimizing system efficiency and to monitor any developing troubles along with overheating or voltage fluctuations at an early stage, making sure that the SPV and FPV perform properly.

The IoT paradigm is made of three layers, namely, (a) Data Acquisition: irradiance sensors (TSL2591) (ams OSRAM, Graz, Austria), temperature sensors (DS18B20) (Analog Devices Inc., Wilmington, MA, USA), and voltage sensors (INA219) (Texas Instruments, Dallas, TX, USA); (b) Data Transmission: XBee modules, [45], operating under the Zigbee protocol at 2.4 GHz frequency; and (c) Cloud Analytics: AWS IoT Core, [46], is utilized for the real-time dashboard visualization. The sensor data is sampled every 5 min, setting thresholds to trigger alerts for overheating and voltage drop. Thus, whenever the temperature rises above 50 °C or the voltage drops below 12 V, the alerts are triggered.

## 4. Results and Discussion

An SPV prototype with a reflector was designed, constructed, and tested alongside an FPV panel under the same conditions for the month of June 2024 at the University of Doha Campus, in Doha, Qatar. Both the SPV and FPV have the same number of solar cells to ensure a fair comparison. In this section, authors will compare the output power and energy of each system, the average measured temperature on the PV cells, and the average measured voltage. Then, the relationship between the measured voltage and temperature of both SPV and FPV is constructed. Finally, both technologies are compared in a table.

### 4.1. Database, Assumption, and Consideration for the Study

**Figure 7:**
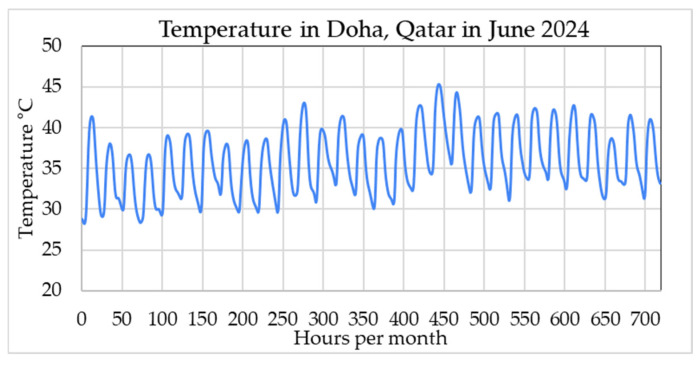
Temperature in Doha, Qatar, in June 2024.

**Figure 8:**
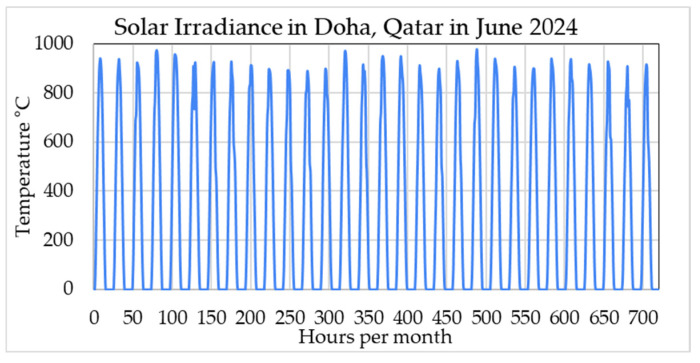
Solar irradiance in Doha, Qatar, in June 2024.

In this paper, authors conducted the experiment and installation of the PV system in June 2024. It is important to mention that the weather in Doha, Qatar, during this period is considered hot and humid. The average ambient temperature during June is around 35.9 °C and can reach 45 °C in some periods. The average solar irradiance is 303.7 W/m^2^ and the maximum is 980 W/m^2^. Figure 7 and Figure 8 present the hourly average temperature and solar irradiance in Doha, Qatar in June 2024.

### 4.2. Output Power and Energy

**Figure 9:**
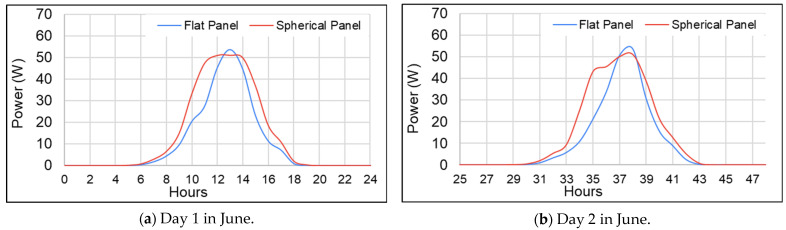

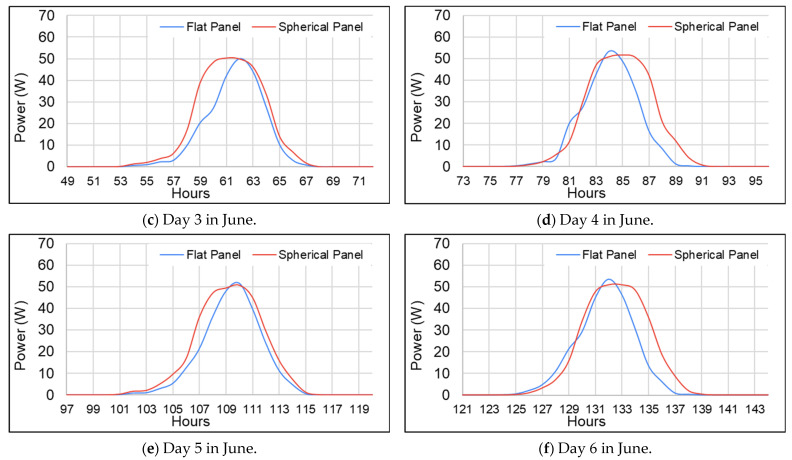
Comparison between the output power of the SPV and FPV over the course of six days in June.

Figure 9a–f present the output power of the SPV and FPV over the first six days in the month of June 2024, respectively. The graph visually demonstrates the key advantage of SPV over FPV in terms of power generation. The power generation is extended during morning, evening, and overcast conditions due to SPV ability to capture low-angle and multidirectional light. While FPV may achieve a higher peak output at solar noon under ideal conditions, SPV typically generates more usable power over the entire course of a day, leading to potentially higher total daily energy production. In conclusion, SPV provides more consistent power over longer daylight hours, reducing reliance on peak noon sun. In addition, SPV is less sensitive to orientation or fixed-tilt angles.

**Figure 10:**
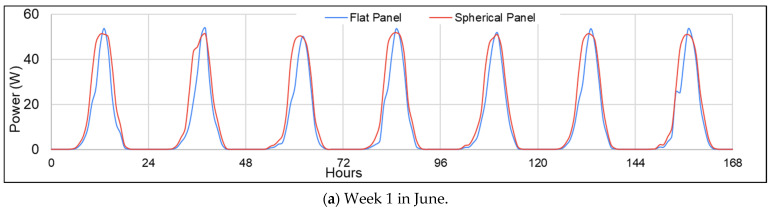

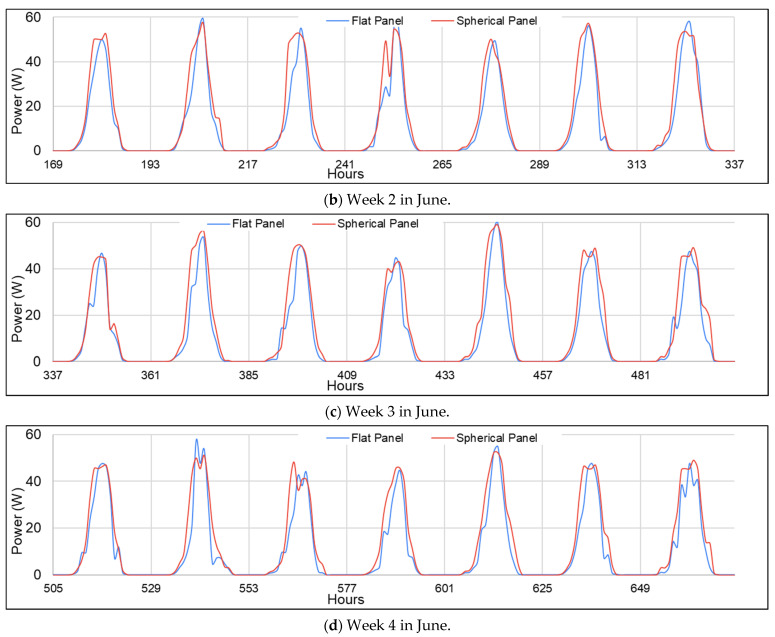
Comparison between the output power of the SPV and FPV over the course of a week.

Figure 10a–d illustrate the power generation of the FPV and SPV systems over the month of June 2024, where each figure corresponds to one week of operation. It can be observed that, at certain times during the day, the measured solar radiation falls below the expected levels, primarily due to transient weather conditions such as cloud cover or partial shading. Qatar’s climate is generally characterized by high temperatures throughout the year; during the summer months, ambient temperatures can reach 45–49 °C, while in winter they may drop to approximately 11 °C. Although rainfall is infrequent, it can occur in any season. In addition, the climate is marked by high relative humidity during summer, particularly in coastal areas such as Doha, due to proximity to the sea.

**Figure 11:**
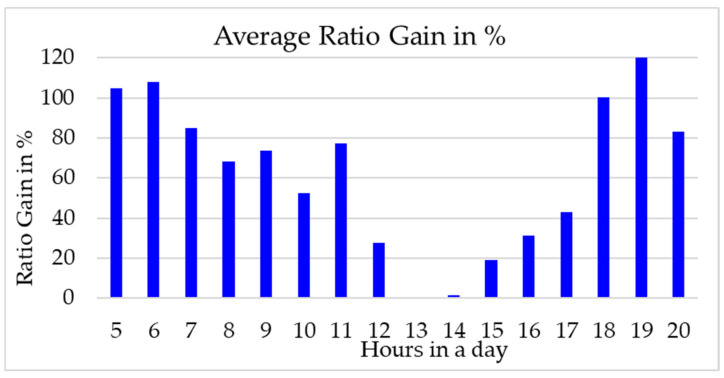
Average hourly gain ratio of the SPV system relative to the FPV system during the month of June 2024.

Figure 11 presents the average hourly gain ratio of the energy generated by the spherical photovoltaic (SPV) system relative to the flat photovoltaic (FPV) system over the month of June 2024. Overall, the SPV system exhibits higher energy generation during the early morning and late afternoon hours compared to the FPV system. In particular, at 5:00 am, the average power generated by the SPV reaches approximately 105% higher than that of the FPV, indicating that the energy output is more than doubled during this period. Around solar noon, between 1:00 pm and 2:00 pm, both systems produce nearly the same average amount of energy. When integrated over the entire month, the SPV system generates more than 26% additional energy compared to the FPV system.

The average output power of the SPV system obtained during the experimental campaign shows a deviation from the theoretically predicted output power determined by Equation (27) when compared to an equivalent FPV system. Based on theoretical modeling, the SPV was expected to generate approximately 46% more energy than the FPV under identical conditions. This expectation is primarily attributed to the spherical geometry of the SPV, which enables improved angular acceptance of incident solar radiation, enhanced utilization of diffuse irradiance, and extended energy capture during early morning and late afternoon hours.

However, experimental results indicate that the SPV system achieved an average energy gain of approximately 26% relative to the FPV with the same number of solar cells. While this represents a substantial improvement over the flat configuration, it remains lower than the theoretical prediction. The discrepancy between theoretical and experimental performance can be attributed to several practical and environmental factors that are not fully captured in idealized models. These include non-uniform irradiance distribution over the spherical surface, partial self-shading effects, thermal losses due to higher surface exposure, gaps between solar cells in the SPV, non-uniform cell performance, and resistive and reflection losses. In addition, experimental operation introduces additional inefficiencies related to manufacturing tolerances, electrical interconnections, and measurement uncertainties. Despite these limitations, the experimentally observed 26% energy gain confirms the fundamental advantage of the SPV concept over conventional flat PV systems, particularly in capturing solar energy during non-peak hours when the incident angle of sunlight is less favorable for flat panels.

Overall, the comparison between theoretical and experimental results highlights the importance of experimental validation for novel photovoltaic geometries. While the theoretical gain represents an upper performance bound, the experimental outcome provides a realistic assessment of achievable benefits under real operating conditions. The observed performance gap also indicates potential avenues for further optimization of SPV design, including improved cell placement, thermal management strategies, and surface treatments to mitigate soiling and optical losses.

### 4.3. Temperature Measurement for SPV and FPV

**Figure 12:**
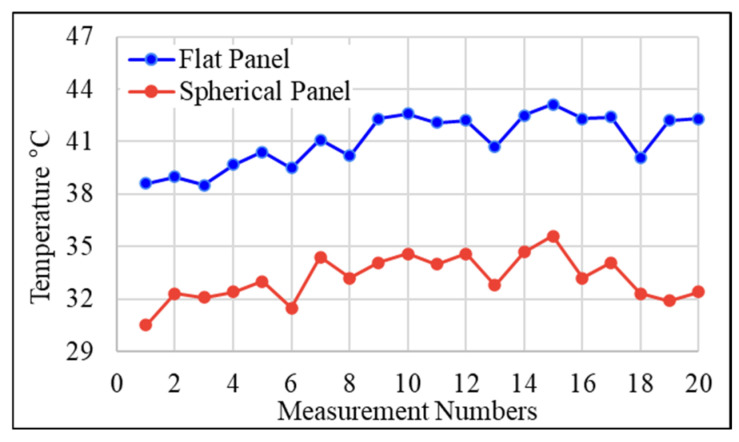
Comparison between the surface temperature on the SPV and FPV for different measurements.

Figure 12 presents the surface temperatures on the FPV and SPV for different measurement samples. This figure clearly demonstrates a significant thermal advantage of the SPV design over the traditional FPV. The spherical shape’s inherent properties promote much better heat dissipation, resulting in consistently lower operating temperatures (typically 5–10 °C cooler). This lower operating temperature directly translates to higher electrical conversion efficiency and better sustained power output, especially during peak sunlight hours, complementing its advantages in capturing light at wider angles as shown previously in Figure 9. The cooler operation is a key factor in the SPV’s overall superior energy yield. Measuring the temperature matters for the following reasons:

Impact on Efficiency: Solar cells become less efficient as their temperature increases. A common rule of thumb is that efficiency decreases by about 0.3–0.5% per degree Celsius rise in temperature above 25 °C (Standard Test Conditions).

Impact on Performance: The hotter solar cell will experience greater efficiency losses due to heat. This contributes to why FPV’s power output drops more sharply around peak sun hours compared to the SPV, even beyond the angle of incidence effects.

Impact on Longevity: Consistently operating at lower temperatures generally reduces thermal stress on materials, potentially leading to longer panel lifespan and reduced degradation rates for the spherical design.

### 4.4. Voltage Measurement for SPV and FPV

**Figure 13:**
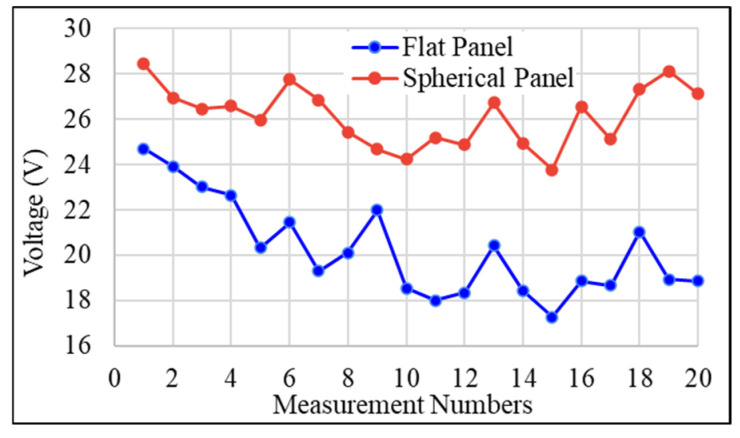
Comparison between the output voltage of the SPV and FPV for different measurements.

Figure 13 demonstrates a significant electrical advantage of the SPV. SPV consistently generates higher operating voltage than the FPV throughout the day. This advantage is directly linked to its superior thermal performance (lower operating temperature), in which the SPV operates at higher voltages at all times. The SPV’s lower operating temperature (Figure 12) enables it to maintain higher voltages (Figure 13), which contributes significantly to its broader and more consistent power output profile (Figure 9), particularly mitigating the sharp power drop seen in the FPV at midday. The thermal advantage is a core enabler of its superior electrical performance. It is important to maintain the operating voltage high for the following reasons:

System Efficiency: Higher operating voltage means less energy loss in wiring ($P_{loss}=RI^{2}$) for the same power output (P = IV).

Inverter Compatibility: Maintaining voltage above an inverter’s minimum operating threshold is crucial. The SPV’s higher/stabler voltage ensures more reliable inverter activation and operation, especially during mornings, evenings, or under partial shading.

Power Output: Voltage is a direct component of power (P = IV). Higher voltage contributes directly to higher potential power output, complementing the spherical panel’s advantage in energy yield.

Battery Charging: For off-grid systems, higher voltage facilitates more efficient battery charging, particularly reaching the required absorption voltage.

### 4.5. Voltage vs. Temperature Measurement for SPV and FPV

**Figure 14:**
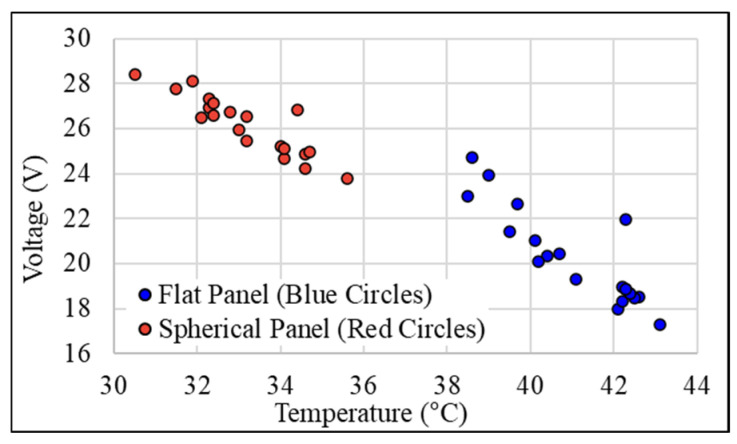
Voltage vs. temperature of FPV vs. SPV.

Figure 14 presents the critical relationship between operating temperature and output voltage for FPV vs. SPV, revealing a key advantage of a spherical design. Both panel types exhibit the fundamental semiconductor property where voltage decreases as temperature increases, but the SPV (red circles) consistently generates higher voltages than the FPV (blue circles) at any given temperature. Crucially, the SPV operates within a cooler temperature range (approximately 30 °C to 36 °C) due to its superior heat dissipation, allowing it to maintain a significantly higher and more stable voltage output (around 24 V to 28 V). In contrast, the FPV operates at hotter temperatures (up to 43 °C), suffering a steeper voltage decline, particularly above 38 °C, resulting in a wider and lower voltage range (approximately 17 V to 25 V). This inherent thermal–electrical advantage explains the SPV’s higher and more consistent power output. Its cooler operation directly preserves voltage, a key component of power (P = IV), minimizing efficiency losses seen in the hotter FPV, especially during peak sunlight.

### 4.6. Comparison Between SPV and FPV

Solar photovoltaic (PV) technology traditionally relies on flat-panel designs, but emerging spherical architectures offer compelling advantages in energy yield, thermal management, and operational stability. While flat panels generate peak power under direct midday sun, their performance declines sharply during mornings, evenings, or suboptimal angles due to fixed orientation and significant temperature-induced efficiency losses [47]. In contrast, spherical panels leverage their geometry to capture diffuse and low-angle sunlight across extended hours, producing a broader, flatter power curve with earlier morning and later evening generation. Crucially, the spherical design’s innate heat dissipation capabilities—attributed to its higher surface-area-to-volume ratio—maintain operating temperatures at 5–10 °C cooler than flat panels. This thermal advantage directly preserves voltage output (24–28 V vs. 17–25 V for flat panels), mitigates efficiency degradation, and minimizes midday power dips. Consequently, spherical panels achieve higher total daily energy output despite potentially lower peak noon power, alongside benefits like reduced thermal stress and enhanced compatibility with power electronics. Table 2 presents a comparison that highlights the advantages and disadvantages of each technology, considering many aspects.

## 5. Conclusions and Future Work

In this paper, authors present an experimental setup of a spherical photovoltaic (SPV) panel and compare it to a flat photovoltaic (FPV) panel with the same number of solar cells for a fair evaluation. This study conclusively demonstrates that the SPV integrated with a paraboloid reflector and IoT-based monitoring system significantly outperforms conventional FPV panel in energy yield, thermal regulation, and operational adaptability. The SPV’s omnidirectional geometry enables consistent capture of direct, diffuse, and reflected irradiance across varying solar angles, extending power generation during mornings, evenings, and suboptimal weather conditions. Experimental results under Qatar’s extreme climate revealed a 32.2% increase in daily energy output compared to FPV, alongside a 14.8–39.7% power gain from the reflector’s light-concentrating capability. Critically, the SPV operated at 8–12 °C cooler than FPV due to superior heat dissipation from its spherical shape, mitigating temperature-induced efficiency losses and sustaining higher voltages (24–28 V vs. 17–25 V). This thermal advantage directly enhanced power stability, particularly during peak irradiance. Additionally, the SPV reduced dust accumulation by 27% and required around 35% less installation area per watt, highlighting its suitability for space-constrained urban environments. While challenges in scalability and manufacturing costs remain, the integration of AI-driven predictive maintenance and dynamic reflectors presents a promising path toward commercial viability. Collectively, this research validates the SPV system as a transformative solution for decentralized solar energy, aligning with global sustainability goals through enhanced efficiency, reliability, and land-use optimization.

Considerations for our future work can be summarized as follows: (a) integrate a sun-tracking reflector, which will increase the reflected solar radiation on the SPV; (b) integrate IoT-based dynamic optimization that helps in adjusting the SPV performance in real time within a closed-loop architecture that accepts data from irradiance, temperature, and voltage sensors; (c) use cutting-edge computing and machine learning models on AWS IoT Core, allowing the tilt angle of the reflector to be dynamically adjusted by servo motors; (d) add a cooling source when the temperature rises above 45 °C in order to increase the efficiency of the solar cells; (e) add dust sensors that trigger an automated cleaning cycle in case of dust accumulation; (f) add an alarm system that sends warning signals when the voltage and temperature of the SPV reach certain thresholds.

## Figures and Tables

**Table 1:** Selected materials for the design and implementation of the SPV.

Materials	Dimension and Description
photovoltaic cells	52 mm × 78 mm, Polycrystalline silicon, 0.7 W peak
wooden shaft	Length: 30 cm
Styrofoam ball	Diameter: 30 cm
Sensors and Data Acquisition	Irradiance sensor (TSL2591) Voltage sensor (INA219) Temperature sensor (DS18B20)
Cloud Analytics	AWS IoT Core (Amazon Web Services IoT), based in USA
Data Transmission and wireless transceiver	XBee modules operating under the Zigbee protocol at 2.4 GHz frequency

**Table 2:** Comparison between FPV and SPV.

Aspect	Flat Solar Panels	Spherical Solar Panels
**Technical Comparison**		
**Light Capture**	Fixed angle; efficiency drops if not aligned with the sun’s position.	Omnidirectional light capture (direct, reflected, ambient).
**Energy Output**	Lower total energy yield, highly dependent on panel orientation and solar incidence angle.	Higher overall energy yield due to multidirectional light capture; experimentally observed gains of ~26% over FPV.
**Angular Response**	Limited angular response; optimal performance near solar noon.	Excellent angular acceptance; effective during early morning and late afternoon.
**Diffuse Radiation Utilization**	Less effective in capturing diffuse radiation.	Efficient utilization of diffuse and reflected irradiance.
**Tracking Requirement**	Often requires single- or dual-axis tracking to improve yield.	Does not require solar tracking due to spherical geometry.
**Performance at Low Sun Angles**	Strong performance under oblique solar incidence.	Significant performance drop at low sun angles.
**Efficiency**	15–22% under optimal conditions; lower in non-ideal angles or diffuse light.	Up to 20% (Sphelar) or higher in prototypes (e.g., Saudi design: +100% with reflectors).
**Heat Dissipation**	Overheats when installed flat; reduced airflow affects efficiency.	Better heat dissipation due to shape; reduces efficiency loss from overheating.
**Temperature Effects**	More predictable thermal behavior and easier cooling strategies.	Larger exposed surface may increase thermal losses if not well managed.
**Dust Resistance**	Prone to dust accumulation, requiring frequent cleaning.	Spherical shape minimizes dust retention; self-cleaning in some designs.
**Space Requirements**	Requires tracking systems or tilting for optimal output; more land for fixed setups.	Compact and flexible; suitable for curved surfaces and urban integration.
**Design Flexibility**	Rigid and flat; limited to fixed installations.	Can be embedded in windows, devices, or 3D structures; transparent or semi-transparent options.
**Economic Comparison**		
**Initial Cost**	Lower upfront cost due to mature manufacturing.	Higher production costs (complex spherical silicon crystallization).
**Maintenance Cost**	Higher if trackers or cleaning systems are needed.	Lower maintenance (no trackers, reduced cleaning needs).
**Land-Use Efficiency**	Lower energy density per unit land area.	Higher energy yield per footprint area
**Scalability**	Easily scalable for utility-scale projects.	Limited scalability due to niche manufacturing processes.
**LCOE (Levelized Cost)**	Lower LCOE for utility-scale installations (e.g., Erthos’ Earth Mount).	Potentially competitive with automation, but currently higher.
**Advantages vs. Disadvantages**		
**Advantages**	Proven technology with widespread adoption.	Omnidirectional energy capture.
	Lower degradation rates in some designs (e.g., Erthos’ flat systems).	Versatile integration (e.g., BIPV, IoT devices).
	Suitable for large-scale farms with tracking systems.	Reduced material waste during production.
**Disadvantages**	Efficiency loss in non-optimal angles/seasons.	High production costs and scalability challenges.
	Vulnerable to shading and soiling.	Lower market penetration and limited field testing.
	Limited aesthetic appeal in urban settings.	Requires reflective backgrounds for peak performance.

## Data Availability

The data presented in this study are available from the corresponding author upon reasonable request.
